# Functional Diversity of p53 in Human and Wild Animals

**DOI:** 10.3389/fendo.2019.00152

**Published:** 2019-03-12

**Authors:** Yi Li, Meng-Chen Zhang, Xiao-Kang Xu, Yang Zhao, Chatoo Mahanand, Tao Zhu, Hong Deng, Eviatar Nevo, Ji-Zeng Du, Xue-Qun Chen

**Affiliations:** ^1^Division of Neurobiology and Physiology, Department of Basic Medical Sciences, School of Medicine, Zhejiang University HHangzhou, China; ^2^Department of Biology, University of Rochester, Rochester, NY, United States; ^3^Department of Pathology, Sir Run Run Shaw Hospital, Zhejiang University, Hangzhou, China; ^4^Department of Pathology and Pathophysiology, School of Medicine, Zhejiang University, Hangzhou, China; ^5^Institute of Evolution and International Graduate Center of Evolution, University of Haifa, Haifa, Israel; ^6^Key Laboratory of Medical Neurobiology of the Ministry of Health, Institute of Neuroscience, School of Medicine, Zhejiang University, Hangzhou, China; ^7^Key Laboratory of Medical Neurobiology of Zhejiang Province, Institute of Neuroscience, School of Medicine, Zhejiang University, Hangzhou, China

**Keywords:** p53, mutation, metastasis, transcriptional factor, stress, variation

## Abstract

The common understanding of p53 function is a genome guardian, which is activated by diverse stresses stimuli and mediates DNA repair, apoptosis, and cell cycle arrest. Increasing evidence has demonstrated p53 new cellular functions involved in abundant endocrine and metabolic response for maintaining homeostasis. However, *TP53* is frequently mutant in human cancers, and the mutant p53 (Mut-p53) turns to an “evil” cancer-assistant. Mut-p53-induced epithelial-mesenchymal transition (EMT) plays a crucial role in the invasion and metastasis of endocrine carcinomas, and Mut-p53 is involved in cancer immune evasion by upregulating PD-L1 expression. Therefore, Mut-p53 is a valuable treatment target for malignant tumors. Targeting Mut-p53 in correcting sequence and conformation are increasingly concerned. Interestingly, in wild animals, p53 variations contribute to cancer resistant and high longevity. This review has discussed the multiple functions of p53 in health, diseases, and nature evolution, summarized the frequently mutant sites of p53, and the mechanisms of Mut-p53-mediated metastasis and immune evasion in endocrine cancers. We have provided a new insight for multiple roles of p53 in human and wild animals.

## Introduction

Since 1979, 40 years of research on p53 have shown it regulates complex and adaptable target gene networks to control broad biological processes (1–3). p53 was first discovered as a 53 kDa protein in SV40-transformed cells (1, 2). p53 protein has five discrete domains with different functions: two N-terminal transactivation domain (TAD) involved in the recruitment of transcriptional co-factors, a central sequence-specific DNA-binding domain (DBD) directly interacting with DNA, a C-terminal oligomerisation domain (OD), and a tetramerization domain (TET) that mediated p53 working in a tetramer manner (4). p53 directly binds to p53 response element (p53REs) that consist of two copies of a 10 base pair motif with the consensus 5′-RRRCWWGYYY-3′ (R = A/G, W = A/T, and Y = C/T) (5, 6). Because of the high diversity of p53REs, p53 controls abundant sets of target genes (3). Initially, p53 is defined as a tumor suppressor, induces cell cycle arrest, apoptosis, and senescence programs to maintain genome integrity in response to genotoxic and oncogenic stresses via regulating target gene expression, such as *Cdkn1a, Puma*, and *Noxa* (3, 7, 8). Recently, novel functions of p53 have been found in regulating metabolism (9–11), stemness (12), antioxidant (13), autophagy (14, 15), ferroptosis (16), differentiation (17), and embryo implantation (18)/fitness selection (19) processes. p53 is frequently mutant in human cancers, which induces aggressive tumors proliferation, invasion, metastasis and immune evasion (20–23). The *TP53* mutation is listed as a potential candidate in disease risk prediction, personalized, and prognostic treatment (24, 25). Wild animals harbor p53 variations (same as mutants in human cancers) that contribute to anticancer and environmental adaptation, which may provide insights into understanding targeting p53 (26–28).

### p53 Is a Cellular Sensor for Homeostasis

p53 is a crucial regulation hub in many physiological processes including metabolism (10), autophagy (14), apoptosis (29), and cell arrest (5) that maintain normal cellular homeostasis (30). Its target genes also play important roles in these processes, for instance, *Glut1/4* (glucose transporter type 1 and 4), *Pltp* (phospholipid transfer protein), and *Dhrs3* (dehydrogenase/reductase 3) are involved in the formation and transfer of lipid in mice liver (10); *Lpin1* facilitates fatty acids oxidation (*FAO*) in low glucose environment but suppresses in normal conditions (31); *Dram* (damage-regulated autophagy modulator 1) and *Aen* (apoptosis enhancing nuclease) promote autophagy, while *Tiger* (*TP53*-induced glycolysis and apoptosis regulator) inhibits autophagy in colon carcinoma cell line (14). In individual cells, the levels of p53 protein exhibit dynamic accumulation with distinct pulses after DNA damage, both p53 pulses and target genes mRNA half-life lead to diversified expression pulses of genes (32, 33). A mathematical model has been proposed to predict the expression patterns of target genes under different p53 inputs, which provides a better understanding of p53 and its target genes interaction (34). These p53 dynamics can control cell fates. Fast-accumulating p53 can reach apoptosis threshold level and triggers cell apoptosis, whereas cells with slow-accumulating p53 are unable to execute apoptosis because they cannot exceed the threshold that increases with time by the activation of anti-apoptotic genes (32, 35), this suggests that the dynamics of p53 and target genes may impact cellular homeostasis.

Furthermore, p53 activates mTOR (downstream mammalian target of rapamycin) and AMPK (AMP-activated protein kinase) signal pathways, indirectly modulates lipid anabolism/catabolism (10, 11), carbohydrate metabolism (11), and autophagy (15). But, AMPK triggers p53-S15 phosphorylation as an upstream activator in response to glucose deprivation in primary mouse embryonic fibroblasts (MEFs) (11). p53 is an obesity regulator, lack of p53 increases lipid accumulation by restraining aromatase expression, which leads to high testosterone levels and obesity in male mice (30). In agouti-related peptide (AgRP) neurons of mice hypothalamus, knockout *TP53* promotes food-induced adiposity and decreases the thermogenesis in brown adipose tissue but overexpressed p53 results in more body weight loss than *TP53* KO mice, and c-Jun N-terminal kinase (JNK) is indispensable in both processes (36).

### Mechanism of p53 in Endocrine Diseases

#### Wt-p53 Involves in Endocrine Diseases

Endocrine and metabolic disorders are common but complex, which has aroused much concern. There is evidence that wild-type p53 (Wt-p53) involves diabetes, liver steatosis and endocrine tumors (30, 37, 38). Wt-p53 impairs insulin secretion signals in pancreatic β cell via inhibiting autophagic clearance in damaged mitochondria of diabetes (38). In type 2 diabetes, Wt-p53 upregulation leads to β cell failure in hyperglycemia and congenital hyperinsulinism, and promotes insulin resistance in adipose tissue (37, 39). Furthermore, Wt-p53 is associated with male-related tumors, the C-terminal lysine methylation of Wt-p53 repressed its transcriptional activity upon DNA damage and prevented cycle arrest in testicular germ cell tumor (40). Androgen restricts Wt-p53 function and causes p53 trans-localization by activating p53 SUMOylation in prostate cancer (41).

#### Mutation Sites in DBD of p53 Occurred in Endocrine Cancers

**Figure 1:**
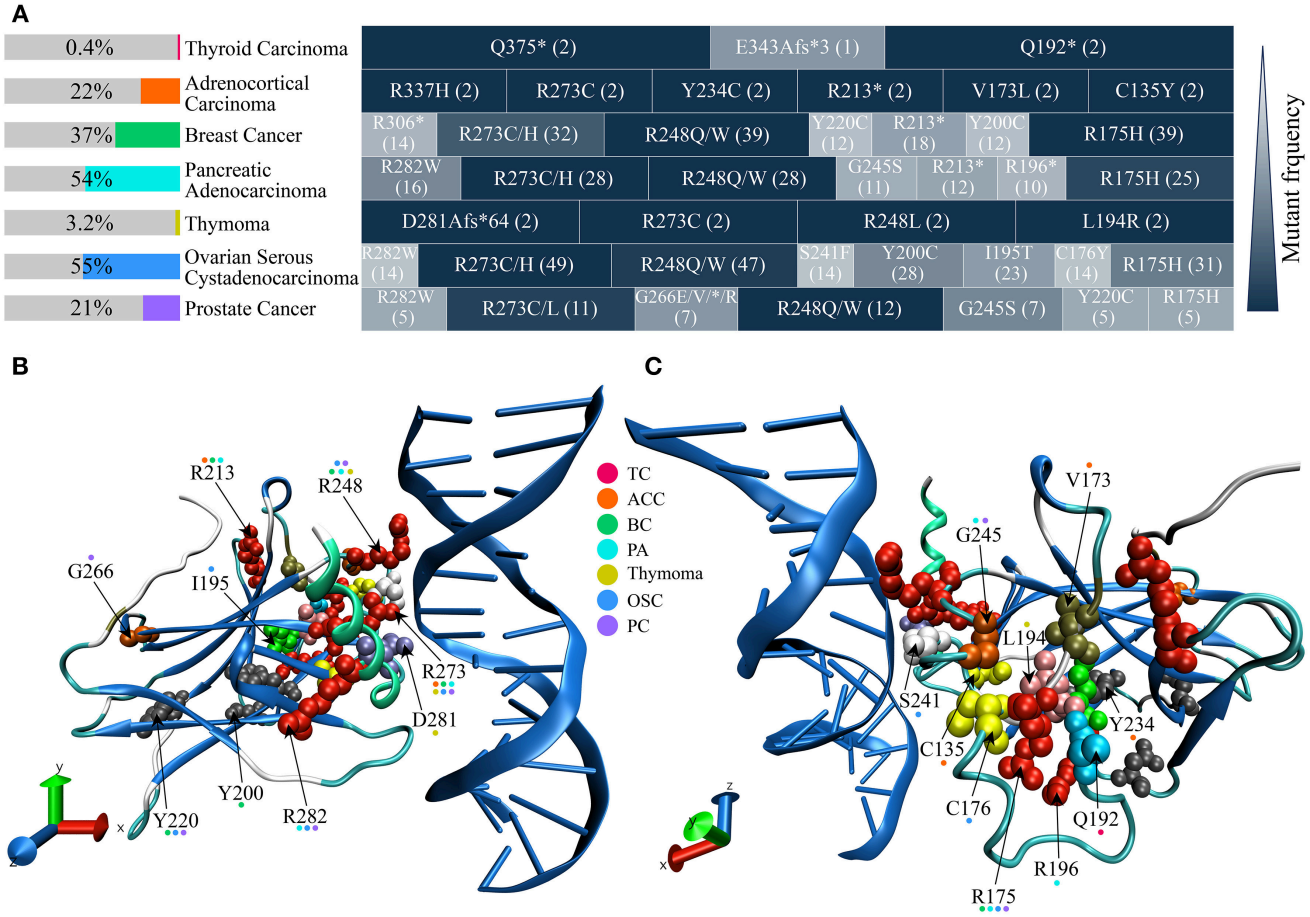
The frequency of p53 mutation, mutated sites, and site location in 3D structure from common endocrine cancers. p53 missense mutation data are obtained from The Cancer Genome Atlas (TCGA) in cBioPortal (http://www.cbioportal.org/). **(A)** p53 mutant frequencies and high frequency mutation sites of seven common endocrine cancers. p53 mutant frequencies of each cancer are shown in the left color bar. The right side indicates p53 high frequency mutation sites in every cancer. Different shades of block depicting the relative mutant frequency of p53 site, compared with the highest mutant rate site (dark blue) in the same cancer. Cases number of respective mutation is shown in parenthesis. **(B,C)** Mutation sites structural position analysis of p53 DBD. Cancers involved in each mutant site are indicated in color dot above AA mark. DBD, DNA binding domain; TC, Thyroid Carcinoma; ACC, Adrenocortical Carcinoma; BC, Breast Cancer; PA, Pancreatic Adenocarcinoma; OSC, Ovarian Serous Cystadenocarcinoma; PC, Prostate Cancer.

Genomic data from more than 20,000 patients have confirmed that the *TP53* is the most commonly mutated gene in all human cancers (42–44), such as hepatocellular carcinoma (45), colorectal cancer (46), lymphoma (47), and mucosal melanoma (48). Data in this review has collected seven endocrine cancers data from The Cancer Genome Atlas (TCGA) in cBioPortal (http://www.cbioportal.org/) (49, 50), and analyzed amino acid (AA) sites of Mut-p53 in these endocrine carcinomas (Figure 1). The frequency of p53 mutations in endocrine cancers is varying, it is ~55% in pancreatic adenocarcinoma and ovarian serous cystadenocarcinoma, only 0.4% in thyroid carcinoma and 3.2% in thymoma (Figure 1A). R175, R213, Y220, R248, R273, and R282 of p53 DBD (residue 98-289) are frequently mutant AA sites in these endocrine cancers, and it is striking that R273 site is mutant in most of the endocrine cancers (Figure 1A). Atlas of location for collected mutated sites from endocrine cancers is displayed in 3D-p53 structure (Figures 1B,C). *TP53* mutations show a preference for missense mutations rather than frameshift or non-sense that are frequently occurred in other tumor suppressor genes (42). From more than 80,000 human cancer cases in the Universal Mutation Database (UMD) (http://p53.fr/the-database), over 70% *TP53* are missense mutations (51, 52). p53 with point mutations commonly produce a full-length protein with one single AA substitution (53). The spectrum of p53 missense mutations contains over 2,000 different AA changes, which affect the interaction between p53 and DNA (42). R175, G245, R248, R273, and R282 are part of hotspot mutations that causing DNA binding loss, which can be divided into two categories: “DNA-contact mutation” (R248 and R273) that contact DNA directly and “conformation mutation” (R175, G245, and R282) that perturb the structure of DBD (42, 53, 54).

Mut-p53 can stably accumulate from escaping the degradation of negative regulators and forming aggregates with p63 and p73 (55, 56).There are three types of p53 mutation consequence, loss-of-function (LOF), dominant-negative (DN), and gain-of-function (GOF), have been concluded (55). First, LOF-p53 abolish the transcriptional activation of partially innate target genes, such as *mdm2* (murine double minute 2), *puma*, and *p21* (42). Second, tetramer Mut-p53 exert a DN effect on Wt-p53 (42). Third, GOF-p53 can regulate novel target genes and turn to be an oncogenic role in inducing tumorigenesis, tumor proliferation, invasion and metastasis, tumor inflammation, tumor tissue remodeling, and evading growth suppresses (55, 57). Moreover, these consequences can occur simultaneously (55).

#### Mut-p53 Mediates Invasion, Metastasis, and Immune Evasion

**Figure 2:**
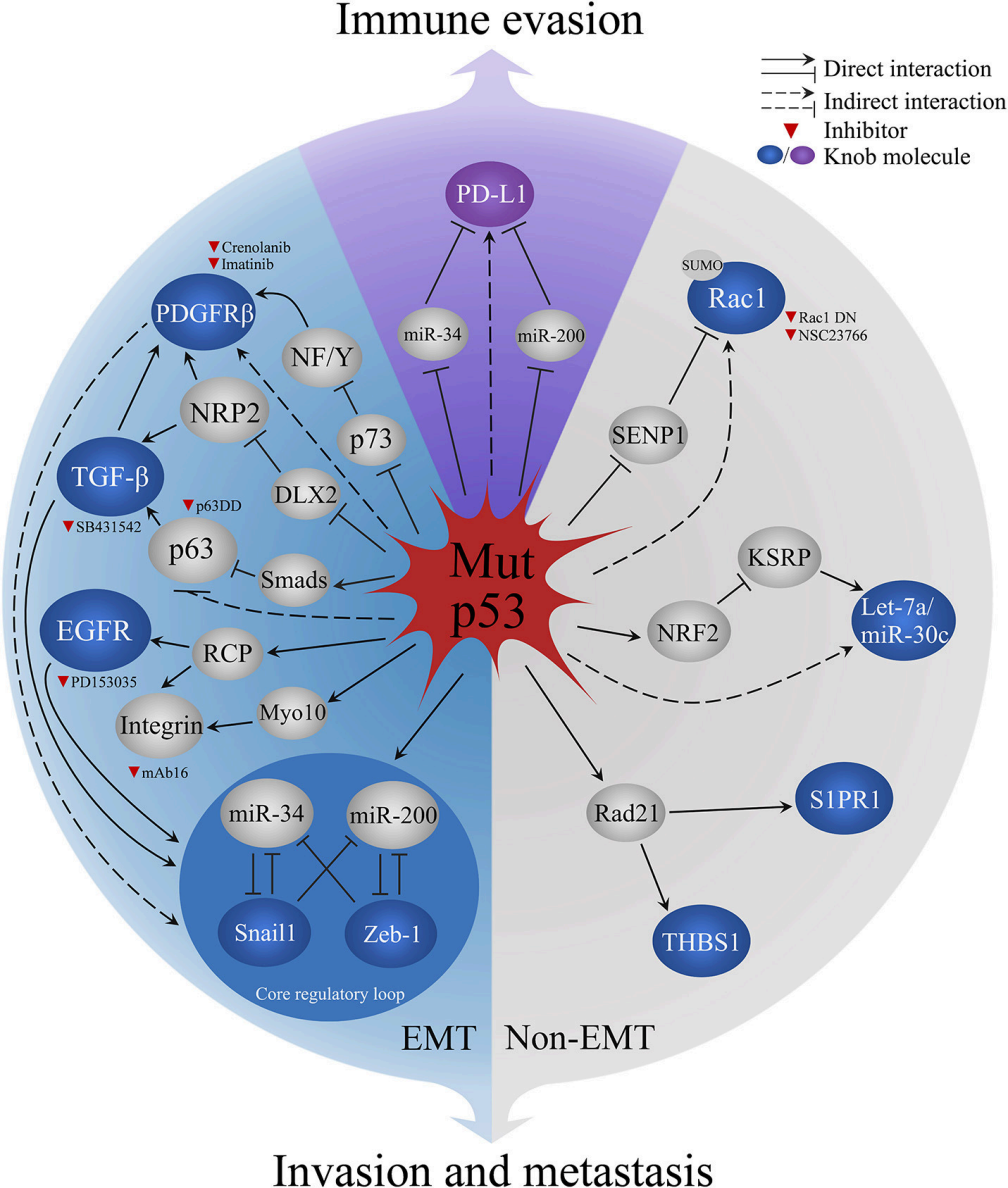
Mechanisms of Mut-p53 aggravate malignant behaviors in endocrine cancers. Blue sector represents Mut-p53-mediated EMT process. Gray sector represents Non-EMT metastasis. Purple sector indicates Mut-p53-regulated PD-L1 immune evasion in cancer cells. Red triangle indicates the inhibitors for current research. PDGFRb, platelet-derived growth factor receptor b; NF/Y, nuclear factors; NRP2, Neuropilin-2; DLX2, Distal-less homeobox 2; TGF-β, transforming growth factor β; EGFR, Epidermal growth factor receptor; RCP, Rab-coupling protein; Myo10, Myosin-X; Rac1, Ras-related C3 botulinum toxin substrate 1; SENP1, SUMO-specific protease 1; miR-34, miRNA 34; miR-200, miRNA 200; NRF2, Nuclear factor-like 2; miR-30c, miRNA 30c; KSRP, KH-type splicing regulatory protein; Rad21, Double-strand-break repair protein rad21; S1PR1, Sphingosine-1-phosphate receptor 1; THBS1, Thrombospodin 1; PD-L1, programmed death-ligand 1.

High invasion and metastatic risk are the fatal hallmark of endocrine adenocarcinomas, such as 60~80% breast and prostate cancer have developed bone metastasis (58), about 70% pancreatic cancer patients die from extensive metastatic diseases (59). Epithelial-mesenchymal transition (EMT) is prerequisite for primary endocrine adenocarcinomas metastasizing to blood, bone, and other organs (58, 60), Mut-p53 is essential to EMT process. This review summarized the pivotal molecular pathways and inhibitors in Mut-p53-mediated EMT/non-EMT process related invasion and metastasis (Figure 2).

Mut-p53 mediates the functions of EMT inducers, including three crucial inducers, transforming growth factor-β (TGF-β), platelet-derived growth factor receptor (PDGFR), and epidermal growth factor receptor (EGFR) (Figure 2). Mut-p53 empowers TGF-β to trigger metastasis of breast cancer cells via forming the ternary complex of Mut-p53, p63, and Smads that prevents the inhibition function of p63 to TGF-β (61). In pancreatic cancer mouse model (KPC model, LSL-Kras^G12D^; P53^R172H/+^; Pdx1-cre), PDGFRβ is upregulated and induces tumor metastasis by Mut-p53-mediated p73/NF-Y complex disruption (62). Furthermore, neuropilin-2 (NRP2) is a co-receptor of TGF-β receptor and PDGFR, which can be upregulated by Mut-p53 R273H and enhance the EMT inducing signals in breast cancer cells (63). Mut-p53 promotes the sustained activation of EGFR via suppressing miR-27a/EGFR axis in breast cancer cells, which increases the mRNA of Zeb-1 and Slug to promote EMT (64, 65). Moreover, Mut-p53 (R175H and R273H) enhance the recycling of integrins and EGFR via rab-coupling protein (RCP), which results in the activation of EGFR/integrin signaling that leads to the invasion behavior *in vivo* and *in vitro* (53). The recycled integrin move to an invasion membrane protrusion to drive cell migration by binding filopodia-inducing motor protein Myosin-X (Myo10) in a Mut-p53 dependent manner (66). Mut-p53 also controls the expression of crucial EMT regulators (Figure 2), including EMT-inducing transcription factors (EMT-TFs; like Snail, Twist, Zeb1, and Zeb2) and post-transcriptional regulators micro-RNA200 (miR-200) and micro-RNA34 (miR-34). Mut-p53 breaks the balance of EMT core regulatory mechanism, two inhibitory loops (Zeb1/miR-200 or Snail1/miR-34), via losing the activity of binding and promoting miR-200/miR-34, which causes the upregulation of Zeb-1/Snail1 that activates the EMT program (67, 68). But, the role of EMT-TFs in metastasis of endocrine carcinomas was challenged recently. Fischer et al. (69) and Zheng et al. (70) have shown the overexpression of miR-200 or depletion of Snai1/Twist1 had no effect on metastasis in breast-to-lung metastasis models and KPC model. However, the depletion of Zeb-1 significantly reduced tumor metastasis in the same KPC model (71), which suggested various EMT-TFs have different sub-functions of tumor metastasis in cancers.

Mut-p53 aggravates malignancy of tumor in the non-EMT mechanism (Figure 2). Mut-p53 competes with SUMO-specific protease 1 (SENP1) to combine Rac1, a small GTPase, and sustains its SUMOylation to promote tumor progression in two breast cancer cell lines [SK-BR-3 (R175H) and MDA-MB468 (R273H)] (72). Mut-p53 (R248) is overexpressed in ovarian cancer cell lines, which can interact with double-strand-break repair protein rad21 (Rad21) and upregulate the expression of Rad21 target genes, sphingosine-1-phosphate receptor 1 (S1PR1), and thrombospodin 1 (THBS1), that related to cell migration in intact cells or cancer cell lines (73). Furthermore, Mut-p53 interacts with nuclear factor-like 2 (Nrf2), a proteasome activator, resulting in the proteasome-mediated degradation of KH-type splicing regulatory protein (KSRP, miRNA maturation factor) in triple-negative breast cancer cells. Thus, downregulated tumor suppressive miRNAs let-7a and miR-30c lead to tumor metastasis (74). There is growing evidence to support that mesenchymal-epithelial transition (MET), the reverse process of EMT, is associated with metastatic colonization (75). Current research suggests reactivating cell signaling pathways and facilitating attachment to heterologous cells are the mechanism of MET. Blocking MET process has been proven to restrain tumor progression in glioma (76). However, the role of Mut-p53 in MET is unclear. Both EMT and MET are critical for embryos/tissues development and wound healing (77), inhibiting general EMT/MET regulators, such as Zeb1, Snail1, or Twist may have adverse effects in other normal biological process. Therefore, blocking p53 negative properties is an attractive strategy for basic and clinical research.

Increasing evidence has shown that Mut-p53 regulates immune evasion by increasing the expression of programmed death-ligand 1 (PD-L1) in tumor cells (78). Based on the genomic, transcriptomic, proteomic, and clinical cancer database in non-small cell lung cancer and lung adenocarcinoma, patients with *TP53* mutations, or Mut-p53-related EMT phenotypes have higher *PD-L1* mRNA expression and low levels of miR-34/miR-200 (23, 78, 79). Both miR-34 and miR-200 suppress PD-L1 by specifically binding to PD-L1 3′-UTR (80). Mut-p53 decreases the expression of miR-34/miR-200 in EMT, which leads to high levels of PD-L1 in non-small cell lung cancer. However, the higher PD-L1 show a significant benefit to PD-1/PD-L1 blockade therapy, p53 may also be a potential guilder for immunotherapy selecting (23, 79).

#### Editing Mut-p53 Return to Homeostasis

Previous research in targeting p53 mostly is to block p53 accumulation or gain of oncogenic function (81–84). However, correcting Mut-p53 gene sequence and protein conformation are emerging as novel strategies in targeting p53. In re-editing p53 sequence, the CRISPR/Cas9 system is a preferred genetic engineering technique that has a promising prospect (85). CRISPR/Cas9 editing with hydrodynamic injection can specifically trigger *TP53* (229 site indel) and *pten* (125 site insertion) mutation in adult mouse liver and directly induce tumors in 2 months that is similar to phenocopies of the Cre-*loxP* method (86). Recently found CRISPR/Cas9 also successfully modified a genomic fragment as large as 65kb length (*TP53* locus about 20.5kb) in mice zygotes and embryos (87). CRISPR/Cas9 technique already can efficiently relieve disease phenotypes of hereditary tyrosinemia type I (HT-1) in mice by correcting fumarylacetoacetate hydrolase (*FAH*) mutation (causing HT-1) (88). These findings suggested that CRISPR/Cas9 could correct Mut-p53 sequence by replacing with a Wt-p53 functional copy, which is possible to be utilized in clinical research. In modifying the conformation of Mut-p53, various compounds have been reported to reconvert Mut-p53 to Wt-like-p53 structure, such as PRIMA-1 [2,2-bis(hydroxymethyl)quinuclidin-3-one] and APR-246 (PRIMA-1^MET^) (89). Recently found a brief exposure of ZMC1 (NSC319726), one of the Zinc metallochaperones compounds from thiosemicarbazone family, is sufficient to reactivate p53 R175H and exhibit Wt-p53 transcriptional activity with few toxicity (90). ZMC1 raises intracellular Zn^2+^ levels to return p53 R175H mutant (zinc-deficient) to “Wt-like-p53” conformation via the recombination of Zn^2+^ and p53 R175H (91), which induces apoptosis in murine cell lines and significantly increases the median survival of KPC mice model (90, 91). It is surprising that a natural product cruciferous vegetable-derived phenethyl isothiocyanate (PEITC) can reactivate p53 R175H function by restoring Wt-like conformation and stimulating canonical Wt-p53 target genes expression. PEITC is already used in anti-Mut-p53 research and clinical trials (https://clinicaltrials.gov/ct2/results?cond=&term$=+PEITC), which explores a new example of targeting p53 by dietary compounds (92). Therefore, targeting p53 with CRISPR/Cas9 re-editing and conformation remodeling compounds are attractive strategies in translation medicine.

## p53 Is an Imprint Gene in Nature Evolution

Wild animals have numerous p53 variations that contribute to environmental adaptation and cancer resistance, however, these variations also have been found in human cancer, which implies special mechanisms exist behind p53 variations in wild animals (26–28, 93). Israel *Spalax* is an anticancer blind subterranean mole rat, there are two specific p53 variations exist in DBD, K172, and K207, compared with human and mouse sequences (R174/R209 in human). R174 and R209 mutations in human p53 have been found in esophageal carcinoma, uterine cervix tumor, colorectal cancer, and endocrine cancers (breast cancer, pancreatic adenocarcinoma, and ovarian cancer) [Figure 1, (93)]. But, p53 variations in *Spalax* is associated with adaptive evolution, which overactives cell cycle arrest genes (*p21*/*cycG*) and downregulates proapoptosis genes (*puma* and *Noxa*) with no *apaf1* expression (93). And, in two subspecies of *Spalax galili* with sharply divergent abutting ecologies, the different methylation modifications of p53 lead to adaptive regulation of p53 pathway and cell-cycle arrest, which indicates p53 epigenetic changes contribute to sympatric speciation (94). Further research reveals that in response to hyperproliferation, released IFN-β induces a p53-triggered anticancer mechanism via inducing necrotic cell death rather than apoptosis in lung primary fibroblasts of *Spalax judaei* and *Spalax golani* (28). p53 codon 104 variations exist in underground mole rat *Myospalax baileyi* (*M.b*), and root vole *Microtus oeconomus* (*M.o*) in Qinghai-Tibet plateau of China, which is also found in fishes and giant tortoise (26, 95). The corresponding human mutation S106 was discovered in one multiple primary cancer case. These specific p53 variations in *M.b* and *M.o* display an extreme environmental adaptation strategy in transcriptional regulation, for instance, *M.b* p53 elicits increased expression of proapoptotic genes under hypoxia/cold and antiapoptotic genes under acidic stress, but *M.o* suppresses all the apoptotic genes and displays a remarkable sensitive to hypoxia (26). In naked mole rat (*Heterocephalus glaber*, NMR), p53 with four PXXP (P = proline, X = any amino acid) motifs in proline-rich domain (five motifs in human and one in rat) increase the convergent evolution possibility of NMR and human, which evolved enhanced DNA damage response and extended lifespan (96). The African and Asia elephant genomes contain a single *TP53* gene and 19 *TP53* retrogenes (1 copy *TP53* in human) (27). The TP53RTG proteins are encoded by 14 retrogenes, and W23G variation exists in all TP53RTGs. TP53RTGs escape MDM2-medicated ubiquitination by the interaction breaking of TP53RTGs and MDM2. Meanwhile, p53 is stabilized by forming TP53RTG/p53 dimer that blocks the degradation by MDM2 (97). High levels of p53 upregulate a re-functionalized retrogene, leukemia inhibitory factor 6 (LIF6), that induces apoptosis in Asian elephant dermal fibroblasts cells (98). The multicopy *TP53*, variations in 14 TP53RTGs, and retrogene reactivation by Wt-p53 are part of the outcome in the evolutionary selection, which enhances the sensitivity in responding to DNA damage, and induces apoptosis rather than DNA repair in elephant (27, 97). Wt-p53 in wild animal elephants and mole rats induces entirely wipe out injured cells by necrosis/strong apoptosis rather than DNA repair like human. Natural wild animals with high longevity and anti-cancer provide an environmental adaptation and cellular homeostasis model for exploring the mechanisms of molecular variations and evolution.

## Conclusion

This mini-review summarizes various roles of p53 in human and animals. As a cellular sensor for homeostasis, p53 involves in metabolism, autophagy regulation, insulin resistance or secretion, and food intake. Wt-p53 is also responsible for the occurrence and developing of endocrine diseases and tumors. p53 is frequently mutant in endocrine cancers and Mut-p53 promotes malignant behaviors by mediating EMT and non-EMT in metastasis and upregulating PD-L1 in immune evasion. Recovering Mut-p53 gene sequence and protein structure with CRISPR-Cas9 or dietary compounds already showed a great research value. Mut-p53 is also a potential candidate for PD-L1 immunotherapy selecting and health risk prediction. In wild animals, p53 variations contribute to environmental adaptation and cancer resistance. p53 is a multifunctional molecule, exploring the new p53 functions, investigating the p53 variations in wild animals, and returning the “evil” Mut-p53 to an “angel” in physiological system are fascinating.

## Data Availability

Publicly available datasets were analyzed in this study. This data can be found here: http://www.cbioportal.org.

## Author Contributions

YL, M-CZ, and X-QC drafted the manuscript. X-QC and J-ZD supervised the project and conceived of the student. X-KX, CM, YZ, and EN contributed to manuscript revisions. TZ and HD contributed to discussions and suggestion. YL and M-CZ contributed to figure and manuscript editing.

### Conflict of Interest Statement

The authors declare that the research was conducted in the absence of any commercial or financial relationships that could be construed as a potential conflict of interest.
